# The chemokine receptor CXCR2 contributes to murine adipocyte development

**DOI:** 10.1002/JLB.1A0618-216RR

**Published:** 2018-12-05

**Authors:** Douglas P. Dyer, Joan Boix Nebot, Christopher J. Kelly, Laura Medina‐Ruiz, Fabian Schuette, Gerard J Graham

**Affiliations:** ^1^ Chemokine Research Group, Institute of Infection, Immunity and Inflammation, College of Medical, Veterinary and Life Sciences University of Glasgow Glasgow United Kingdom; ^2^ Wellcome Centre for Cell‐Matrix Research, Faculty of Biology, Medicine and Health, Manchester Academic Health Science Centre University of Manchester Manchester United Kingdom; ^3^ Lydia Becker Institute of Immunology and Inflammation, Faculty of Biology, Medicine and Health, Manchester Academic Health Science Centre University of Manchester Manchester United Kingdom

**Keywords:** adipose, macrophage, neutrophil

## Abstract

Chemokines are members of a large family of chemotactic cytokines that signal through their receptors to mediate leukocyte recruitment during inflammation and homeostasis. The chemokine receptor CXCR2 has largely been associated with neutrophil recruitment. However, there is emerging evidence of roles for chemokines and their receptors in processes other than leukocyte migration. We have previously demonstrated that CXCR2 knockout (KO) mice have thinner skin compared to wild‐type mice. Herein we demonstrate that this is due to a thinner subcutaneous adipose layer, as a result of fewer and smaller individual adipocytes. We observe a similar phenotype in other fat depots and present data that suggests this may be due to reduced expression of adipogenesis related genes associated with adipocyte specific CXCR2 signaling. Interestingly, this phenotype is evident in female, but not male, CXCR2 KO mice. These findings expand our understanding of nonleukocyte related chemokine receptor functions and help to explain some previously observed adipose‐related phenotypes in CXCR2 KO mice.

**Abbreviations**
ACKR: Atypical chemokine receptors
COPD: Chronic obstructive pulmonary disease
FABP4: Fatty acid binding protein 4
GPCR: G‐protein coupled receptor
Het: Heterozygous
PPARγ: Peroxisome proliferator‐activated receptor gamma
vWF: von Willebrand factor
WAT: White adipose tissue
WT: Wild‐type

## 1 INTRODUCTION

Chemokines belong to a family of approximately 50 structurally related proteins that signal through their cognate G‐protein coupled receptors (GPCRs) to mediate cellular migration and recruitment.^1^ Chemokine receptors are expressed on leukocytes at rest and during inflammation and play an important role in the multistep process of leukocyte migration from the circulation, across the endothelium and within tissues.^2^ Chemokine activity is also fine‐tuned by a subfamily of receptors called atypical chemokine receptors (ACKRs), which are stromally expressed and that regulate local chemokine presentation and availability.^3^ Given their central role in leukocyte recruitment, chemokines and their receptors are integral to the immune response and thus inflammatory based diseases.^4^ The chemokine system has therefore been a target for therapeutic intervention in inflammatory disease. However, there has been limited success in this regard^5^, ^6^ and this is thought to be at least partially due to our lack of basic understanding of the breadth and complexity of chemokine function.^6^ Chemokines and their receptors also play a key role in homeostatic processes. For example, the CXCR4 receptor and its ligand CXCL12 are critical to retention and maturation of hematopoietic stem cells in the bone marrow^7^ and other homeostatic chemokine receptors are central contributors to cellular address codes ensuring precise temperospatial leukocyte migration under resting conditions.^6^ In addition to their involvement in regulating cellular migration in immune and inflammatory responses chemokines and their receptors, in association with the ACKRs, display evidence of significant pleiotropy contributing to the regulation of processes such as angiogenesis,^8^ cellular proliferation,^9^ and apoptosis.^10^ Chemokines, their receptors, and the ACKRs also play key roles in development including governing stem cell migration within the embryo and contributing to the regulation of branching morphogenesis.^11^, ^12^

CXCR2 is a chemokine receptor that has largely been associated with expression, and function, on neutrophils during inflammatory responses. Along with its ligands CXCL1‐3 and CXCL5‐8, CXCR2 controls neutrophil release from the bone marrow^13^ and enables their recruitment to inflamed and infected sites.^14^, ^15^ An additional, noncanonical CXCR2 ligand, MIF, has also been identified and is thought to play a role in recruitment of myelomonocytic cells during atherosclerosis.^16^, ^17^ Given the role of neutrophils in propagating inflammatory disease CXCR2 is seen as a viable therapeutic target in a range of pathologies,^18^ for example, chronic obstructive pulmonary disease (COPD)^19^ and chronic pancreatic inflammation.^20^ Furthermore, CXCR2 blockade is also suggested to be of potential use in cancer therapy due to its ability to prevent recruitment of pro‐tumorigenic myeloid derived suppressor cells to the tumor microenvironment.^21^, ^22^

We have previously demonstrated that adult female CXCR2 knockout (KO) mice have thinner skin than wild‐type (WT) mice.^23^ Here we present data explaining the basis for this phenotype. Specifically we show that CXCR2 KO female mice have smaller adipocytes in several different fat depots, possibly because of aberrant expression of CXCR2‐regulated adipogenesis related genes.^24^ Our findings may, at least in part, explain CXCR2 KO mouse protection from obesity‐induced insulin resistance.^25^ Overall the results presented broaden our understanding of the role of the chemokine system in adipose tissue regulation and add to the developmental processes in which chemokines play a role.

## 2 MATERIALS AND METHODS

### 2.1 Mice

CXCR2 KO^15^ (backcrossed for at least 12 generations onto the C57/Bl6 background) and WT mice were bred in a specific pathogen‐free environment and fed a normal lab diet (ad libitum) in the animal facility of the Beatson Institute for Cancer Research and were used at the indicated age, juvenile (6 wk) and adult (8‐12 wk), in accordance with the animal care and welfare protocols approved by the animal welfare and ethical review board at the University of Glasgow. The observed phenotype described herein was also confirmed to be present in littermates. All experiments were performed under the auspices of a United Kingdom Home Office project license.

### 2.2 Histology

Skin and fat depots were harvested from mice, fixed in formalin, processed, and embedded in paraffin wax. A 5‐μm‐thick tissue sections were cut from these paraffin blocks, baked (65°C for 30 min) onto SuperFrost slides (Thermo Scientific, Waltham, MA, USA), dewaxed in xylene, rehydrated, and stained with H&E counterstain. Finally, slides were dehydrated and mounted using DPX (Leica, Wetzlar, Germany).

Myeloperoxidase (neutrophil) and von Willebrand factor (vWF) staining was undertaken by the Diagnostic services unit, School of Veterinary Medicine at the University of Glasgow. Astra blue (Mast cell) and MAC2 (macrophage) staining was undertaken as described in detail previously.^23^ The indicated measurements were then taken from blinded samples using the Zen software platform (Zeiss, Oberkochen, Germany).

### 2.3 Flow cytometry

Skin and fat depots were harvested from resting WT or CXCR2 KO mice, digested and then analyzed for leukocyte content using flow cytometry. Lower dorsal skin for shaved mice was dissected, cut into small pieces, and digested in HBSS containing DNase I (100 μg/ml), collagenase D (1 mg/ml), and dispase II (500 μg/ml) (Roche, Basel, Switzerland) for 90 min with shaking at 37°C. FBS (20 μl) was added to neutralize enzymes and homogenates were filtered through 70 μm filters and washed in PBS. Inguinal and perigonadal adipose tissue was digested as described elsewhere.^12^

Single cell suspensions prepared from digested tissues or in vitro adipocyte cultures were washed into PBS, stained with fixable viability solution (eBioscience, Thermo Scientific) and blocked with FcR reagent (Miltenyi Biotech, Gladbach, Germany). Suspensions were then washed into PBS containing 1 mM EDTA and 1% FBS (FACS buffer) (Sigma‐Aldrich, St. Louis, MO, USA) and stained with antibodies diluted 1:200 in FACS buffer (CD45, F4/80 (eBioscience, Thermo Scientific), CD11b, Ly6G, CD117 (Biolegend, San Diego, CA, USA), Siglec F (BD Biosciences, Franklin Lakes, NJ, USA), and CXCR2 (R & D Systems, Minneapolis, MN, USA)). Samples were fixed and analyzed using a Fortessa flow cytometer (BD Biosciences) based within the Institute of Infection, Immunity and Inflammation flow cytometry core facility at the University of Glasgow.

### 2.4 Adipocyte differentiation, stimulation, and analysis

The 3T3‐L1 fibroblast cell line (a gift from Professor Gwyn Gould, University of Glasgow) was differentiated as described elsewhere,^26^ briefly cells were cultured in DMEM with 10% FCS at 37°C in 5% CO_2_. To induce differentiation, cells were grown to confluence before addition of differentiation medium (DMEM containing 10% FBS (Gibco, Thermo Scientific), 5 μM troglitazone (Tocris, Bristol, United Kingdom), 1 μg/ml insulin (Sigma‐Aldrich), 0.5 mM IBMX (Sigma‐Aldrich), and 0.25 μM Dexamethasone (Sigma‐Aldrich). After 3 days culture, the media was changed to DMEM containing 10% FBS, 5 μM troglitazone, and 1 μg/ml Insulin. Following a further 3 days of culture, media was changed to DMEM containing 10% FBS only and then changed every 2 days. Cells were analyzed 10 days after initiation of differentiation. Differentiation was also undertaken in the presence of the CXCR2 inhibitor 1 (SB 332235) or CXCR2 inhibitor 2 (SB 225002) (10 μM) (Tocris) to determine its effect on this process. Oil Red‐O staining was used to confirm differentiation. Media was aspirated and cells washed with phosphate buffered saline before fixation in 10% formalin solution for 1 h. Monolayers were incubated with 60% Isopropanol for 2 min before staining with Oil Red‐O solution for 5 min and washing with water. Finally, cells were counterstained with hematoxylin and washed before imaging using an EVOS FL auto microscope (Invitrogen, Carlsbad, NM, USA). Cell size and lipid area were calculated from acquired images using FIJI software.^27^ Quantification of Oil Red‐O staining was undertaken as described previously,^24^ following fixation cells were washed with water and stained with 0.5% Oil Red‐O (w/v) in isopropanol for 15 min. Cells were washed with water to remove excess dye before Oil Red‐O was eluted with isopropanol and quantified (OD540) relative to blank wells.

Western blot analysis of cell cultures lysed in RIPA buffer (Thermo Scientific) was undertaken as described elsewehere^24^ using an antiperoxisome proliferator‐activated receptor gamma (PPARγ) antibody (Cell Signaling Technologies, Danvers, MA, USA) and an anti‐GAPDH antibody (Cell Signaling Technologies).

### 2.5 PCR and qRT‐PCR analysis

Samples from fat depots were harvested and briefly stored in RNAlater (Invitrogen) at −80°C. RNA was then extracted from these samples using Qiazol and physically disrupted by shaking with ball bearings in a Tissue Lyser LT (Qiagen, Hilden, Germany) at 50 oscillations per second for 10 min. The fluid phase was transferred to a clean tube and centrifuged at 4°C, 10,000 RCF for 15 min. The fluid phase was aspirated and transferred to a fresh tube prior to standard RNA extraction using the miRNeasy extraction kit (Qiagen). Purified RNA (500 ng) was converted to cDNA using the high capacity RNA to cDNA kit (Thermo Scientific).

cDNA was analyzed for CXCR2 expression using the indicated primers (Table 1), Q5 High‐Fidelity DNA polymerase and DNTP mix (NewEngland Biolabs, Ipswich, MA, USA) using a standard thermocycler programme. The resulting products were “run” on a 2% agarose gel containing ethidium bromide and the gel analyzed using an Alpha Imager (Alpha Innotech, San Leandro, CA, USA), following electrophoresis.

**Table 1:** Primer sequences for qPCR analysis

GENES		Sequence
PPARγ	Forward	5’‐GCCTATGAGCACTTCACAAGA‐3’
	Reverse	5’‐ATCACGGAGAGGTCCACAGA‐3’
GAPDH	Forward	5’‐CAGCAAGGACACTGAGCAAG‐3’
	Reverse	5’‐TATTATGGGGGTCTGGGATG ‐3’
CXCR2	Forward	5’‐TGTCTGCTCCCTTCCATCTT‐3’
	Reverse	5’‐CCATTTCCTCTCCTCCAGCT‐3’
FABP4	Forward	5’‐AAATCACCGCAGACGACAGG‐3’
	Reverse	5’‐GCTTGTCACCATCTCGTTTTCT‐3’
18S	Forward	5’‐GACTCAACACGGGAAACCTC‐ 3’
	Reverse	5’‐TAACCAGACAAATCGCTCCAC ‐3’

For qRT‐PCR, cDNA was analyzed using the indicated primers (Table 1) and SYBR Green FastMix (Quanta bio, Beverley, MA, USA) in a QuantoStudio 7 flex machine (Life Technologies, Carlsbad, NM, USA) and compared to standards for each gene, using the indicated primer sets.^28^ Absolute copy number for each sample was then plotted relative to a house keeping gene control.

### 2.6 Statistical analysis

Data sets containing only two groups were initially analyzed for normal distribution (KS normality test) and equal variance (*F* test) using GraphPad Prism software. Normally distributed data with equal variance were analyzed using an unpaired Student's *t* test, normally distributed data with unequal variance were analyzed using an unpaired *t* test with Welch's correction and nonnormally distributed data with equal variance were analyzed using a Mann‐Whitney test. Data sets containing more than two groups that were all normally distributed were analyzed using a one‐way ANOVA with Tukey's multiple comparison test and data sets containing more than two groups that were not all normally distributed were analyzed using a one‐way ANOVA with Kruskal‐Wallis comparison. Where data were deemed significantly different (*P* < 0.05) actual *P* values are provided in the figures from the indicated statistical test.

### 2.7 Online supplemental material

**Supplemental Fig**. 1. Western blot images for PPARγ and GAPDH expression analysis in differentiated adipocytes, CXCR2 expression on undifferentiated and differentiated cells and viability of cells cultured in the absence or presence of vehicle or CXCR2 inhibitors.

## 3 RESULTS

### 3.1 Smaller adipocytes are associated with a thinner subcutaneous adipose layer in female CXCR2 KO, compared to WT, mice

**Figure 1:**
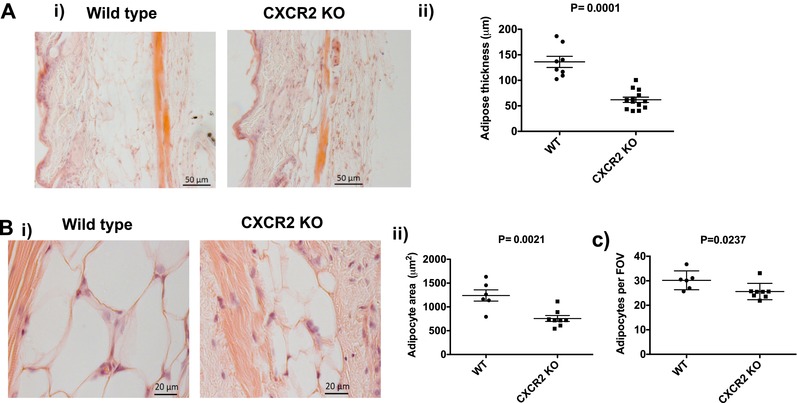
**Female CXCR2 KO mice have a thinner subcutaneous adipose layer due to fewer and smaller adipocytes**. Skin was dissected from adult female mice before processing and H&E staining of sections from wild‐type and CXCR2 KO mice. (A) (i) Brightfield microscopy was used to take images of the skin (scale bars: 50 μm) and (ii) thickness of the adipocyte layer was systematically measured. (B) (i) the subcutaneous adipose depot was further imaged at high magnification (scale bars: 20 μm) and (ii) the size of individual adipocytes measured and expressed as μm^2^. (C) In addition, the number of individual adipocytes contained in the adipocyte layer (per field of view; FOV) was quantified. Data are plotted as mean (±sem) (Aii and Bii) or (±SD) (C) from one experiment containing at least 5 mice in each group, representative of at least 2 separate experiments. Each symbol represents an individual mouse. Data were analyzed with an unpaired *t* test with Welch's correction (i), unpaired *t* test (ii) and a Mann‐Whitney test (iii)

As reported in a previous study, and as shown in Fig. 1, we observed that female CXCR2 KO mice have thinner skin.^23^ Gross histologic (Fig. 1Ai) and quantitative assessment (Fig. 1Aii) of subcutaneous adipose layer thickness in WT and CXCR2 KO female mice revealed that this reduced skin thickness is specifically associated with a thinner adipocyte layer and no significant differences in epidermal or dermal thickness were noted (data not shown). High power magnification revealed that the adipocytes in the CXCR2 KO mouse subcutaneous adipose layer were typically smaller than those in WT adipose tissues (Fig. 1Bi) and this was confirmed by measuring the size of individual adipocytes in the CXCR2 KO and WT mice which showed the CXCR2 KO adipocytes to be significantly (*P* = 0.0021) smaller in size than the WT cells (Fig. 1Bii). In addition to reduction in size, the CXCR2 KO mouse adipose layer contained fewer cells as assessed by counting cells per field of view (Fig. 1C). Overall therefore, these data show that female CXCR2 KO mice have a thinner adipose layer than WT mice and that this relates to smaller, and fewer, adipocytes within the CXCR2 KO adipose layer.

### 3.2 Adipocytes from multiple tissue sources are smaller in CXCR2 KO mice

**Figure 2:**
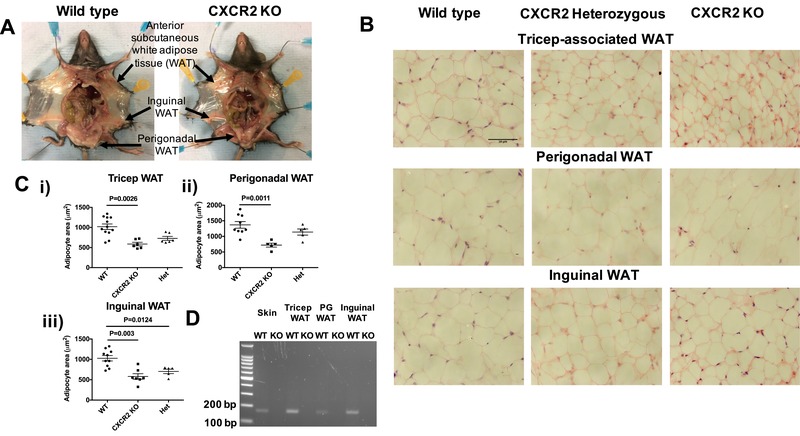
**Adipocytes from multiple sources are smaller in CXCR2 KO female mice, compared to wild‐types**. (A) Mice were dissected to allow visualization of white adipose tissue (WAT) depots at the indicated sites. (B) WAT was dissected, fixed, and processed from each of these 3 areas and sections were stained with H&E (scale bars: 50 μm) from wild‐type, CXCR2 heterozygous (het), and CXCR2 knockout (KO) mice. (C) The area occupied by individual adipocytes was calculated and averaged for each individual mouse, data from each mouse was plotted for (i) tricep; (ii) perigonadal, and (iii) inguinal sites, where each symbol represents an individual mouse. (D) Skin and adipose depots were dissected from WT and CXCR2 KO mice and analyzed for CXCR2 expression by nonquantitative PCR as demonstrated by an expected product of 169 base pairs from the CXCR2 specific primer set. Data from two experiments are pooled and plotted as mean (±sem). Data were analyzed using an ordinary one‐way ANOVA with Tukey's post hoc test **(Cii and iii)** or with a Kruskal‐Wallis test **(Ci)**

Visual analysis of the anterior subcutaneous (tricep‐associated), inguinal and perigonadal white adipose tissue (WAT) reveals that reduced size of adipose depots in CXCR2 KO mice appears to be wide spread (Fig. 2A). Closer histologic analysis of tricep‐associated, perigonadal, and inguinal adipose depots in CXCR2 KO mice revealed, again, that smaller depots were associated with reduced adipocyte size (Fig. 2B) and quantitative analysis revealed this to be a significant (*P* = 0.0026, 0.0011, or 0.003, respectively) reduction (approximately 50%) at each of these tissue sites (Fig. 2Ci, 2Cii, and 2Ciii). Interestingly, CXCR2 heterozygous (het) null female mice display haploinsufficiency in adipocyte size in the inguinal adipose depots, but not in perigonadal and tricep‐associated adipose tissue (Fig. 2C).

Nonquantitative PCR (Fig. 2D) revealed that cells in all the WT fat depots expressed CXCR2 mRNA and that it was completely absent in the CXCR2 KO mice.

### 3.3 Reduced adipose tissue size is seen in female, but not male, CXCR2 KO mice

**Figure 3:**
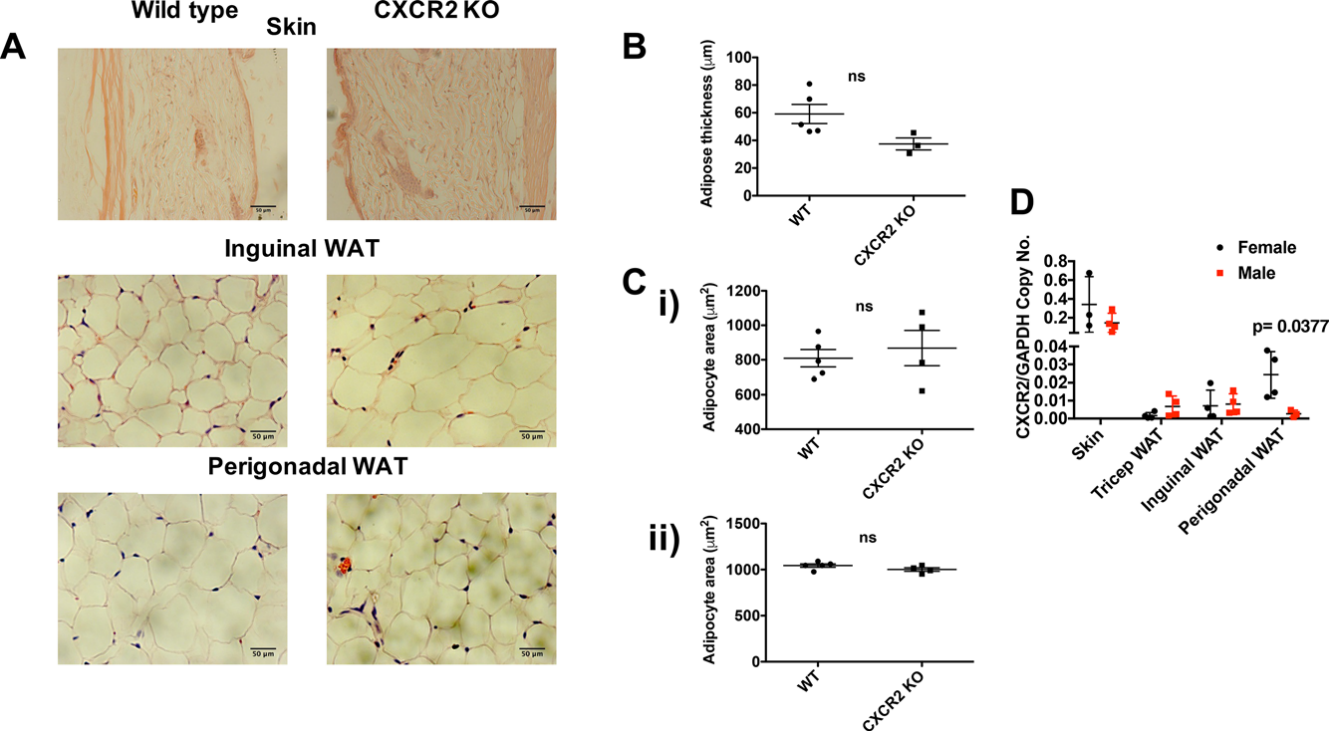
**CXCR2 KO male mice have no significant change in adipocyte size compared to wild‐types**. (A) Skin and adipose depots were dissected from adult male mice (>8 wk) before processing and H&E staining of sections from wild‐type and CXCR2 KO mice. Brightfield microscopy was used to take images of the skin, inguinal adipose, or perigonadal adipose tissue (scale bars: 50 μm). (B) Adipose thickness and (C) individual adipocyte area for (i) inguinal and (ii) perigonadal sites were measured. (D) Quantitative real‐time PCR was used to analyze CXCR2 expression in skin or adipose tissues from male and female mice, relative to the house‐keeping gene, GAPDH. Data are plotted as mean (±sem), where each symbol represents data from an individual mouse and analyzed using an unpaired *t* test or Welch's *t* test (D), ns = not significant

Our initial analyses were performed using female mice. To investigate any potential gender specificity of this phenotype, adult male CXCR2 KO mice were also analyzed, in comparison to WT mice. In contrast to female mice, CXCR2 KO male mice showed no significant differences in subcutaneous adipose thickness or adipocyte size in skin, inguinal, or perigonadal adipose depots. This was apparent on gross histologic assessment (Fig. 3A) as well as on more detailed quantitative analysis which revealed no significant differences in subcutaneous adipose layer thickness (Fig. 3B) and no significant difference in adipocyte size in inguinal or perigonadal sites (Fig. 3Ci and ii). Comparison with the data from analysis of adipose tissue and adipocytes in female mice (Fig. 1) suggests that WT females show generally increased subcutaneous adipose thickness and greater adipocyte size compared to males and that CXCR2 KO females have subcutaneous adipose tissue, and adipocytes, of comparable size to those in WT males of the same age. Taken together these data suggest that CXCR2 plays a role in regulating adipocyte size in female, but not male, mice in resting adipose tissue.

qRT‐PCR analysis of CXCR2 mRNA expression in male or female whole skin, tricep WAT, inguinal WAT, or perigonadal WAT demonstrated that the highest expression was in the whole skin (Fig. 3D). Female mice express significantly higher levels of CXCR2 mRNA in their perigonadal adipose tissue, compared to males.

### 3.4 The subcutaneous adipose layer from CXCR2 KO mice contains comparable levels of myelomonocytic cells to WT mice

**Figure 4:**
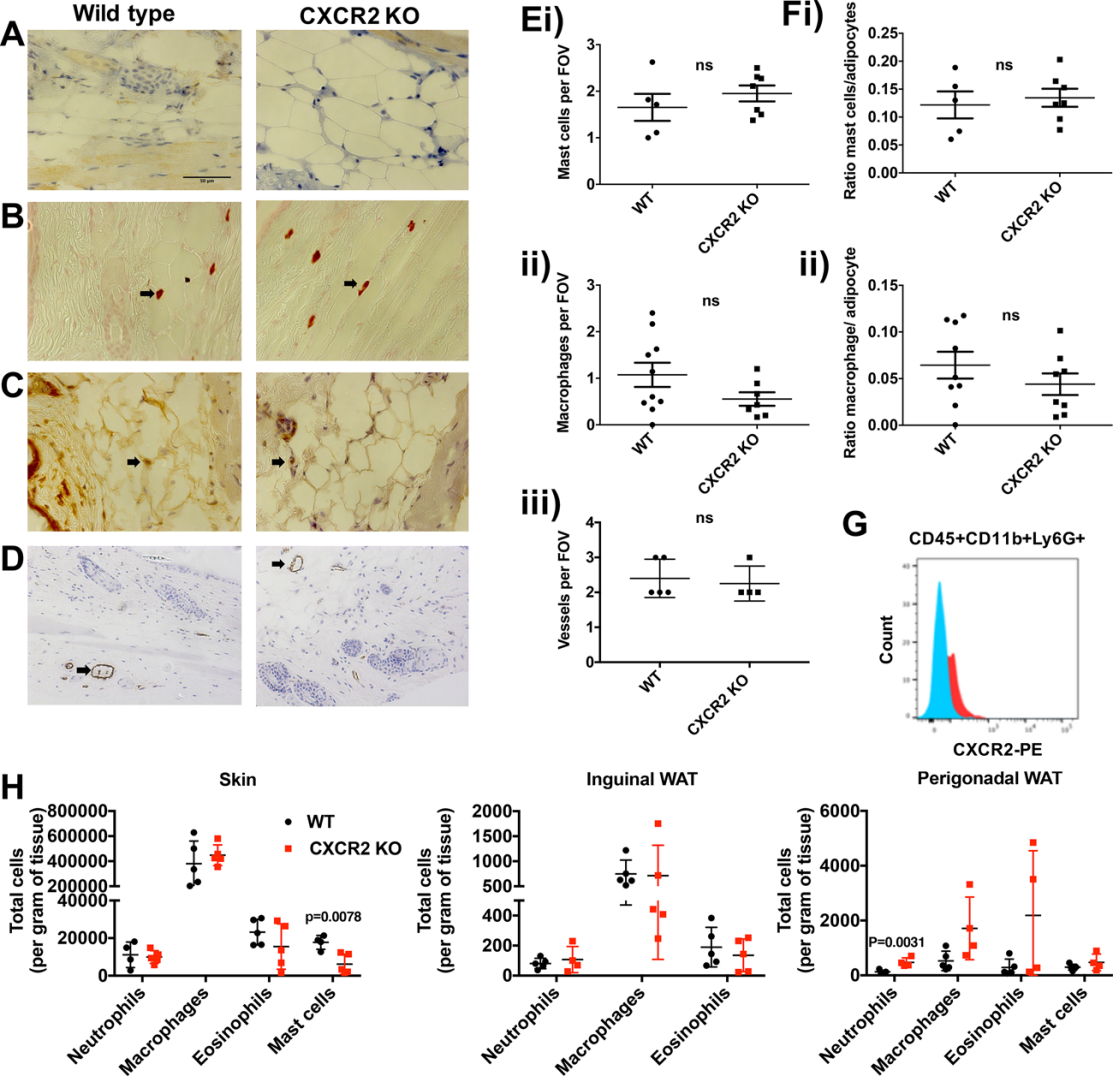
**Resting CXCR2 KO mice show limited differences in neutrophil, mast cell, macrophage, eosinophil or blood vessel numbers in subcutaneous fat**. Skin was dissected from adult female mice before processing and staining (scale bars: 50 μm) to detect (A) neutrophils, (B) mast cells, (C) macrophages, and (D) von Willebrand factor positive vessels from wild‐type and CXCR2 KO mice. (E) The numbers of mast cells (i), macrophages (ii), and blood vessels (iii) were quantified and plotted as the mean number per field of view. (F) (i) Mast cell and (ii) macrophage numbers were also expressed as ratio of cell number to adipocyte number. (G and H) Flow cytometry was used to analyze leukocyte content of skin, inguinal WAT, and perigonadal WAT. (G) CXCR2 positive staining in CD45+CD11b+Ly6G+ cells from skin samples. (H) Quantification of neutrophil (CD45+CD11b+Ly6g+), macrophage (CD45+CD11b+SiglecF‐F4/80+), eosinophil (CD45+CD11b+F480+SiglecF+) and mast cell (CD45+CD117+) content of skin, inguinal WAT, and perigonadal WAT. Data are plotted as mean (±sem), where each symbol represents data from an individual mouse and analyzed using an unpaired *t* test, representative of two separate experiments (A‐F), ns = not significant

The cells traditionally associated with CXCR2 expression, and function, are neutrophils, which are key players in the inflammatory response.^18^ For this reason, and given previous studies describing a link between adipose tissue regulation, chemokine receptors (CXCR2, CCR2, or CX3CR1) and inflammation,^29^, ^30^, ^31^ we determined the presence of neutrophils, mast cells, macrophages, and vWF positive blood vessels within the subcutaneous adipose layer of the skin of WT and CXCR2 KO female mice (Fig. 4). As expected, myeloperoxidase staining clearly demonstrated the complete absence of neutrophils in the subcutaneous adipose tissue of CXCR2 KO mice (Fig. 4A) and this was seen for both female and male (data not shown) mice. Furthermore, Astra blue, Mac 2, and VWF staining demonstrated no visual (Fig. 4B, C, and D) or quantitative (Fig. 4E and F) changes in absolute numbers of mast cells, macrophages and blood vessels, respectively, between WT and CXCR2 KO mice. In addition, the ratio of mast cells, or macrophages, to adipocytes was unchanged between WT and CXCR2 KO female mice (Fig. 4F).

Flow cytometric analysis revealed that the only leukocyte population expressing CXCR2 from the skin, inguinal adipose, or perigonadal adipose were skin neutrophils (Fig. 4G). Flow cytometric analysis of leukocyte content of the skin also revealed no differences in the levels of neutrophils, macrophages, and eosinophils and a significant reduction in mast cells in the CXCR2 KO mice (Fig. 4H). Similar analysis of inguinal and perigonadal adipose tissue demonstrated few differences in leukocyte levels in WT CXCR2 KO mice. The only significant change observed was an increase in neutrophil levels in CXCR2 KO perigonadal tissue.

Together, these data suggest that CXCR2 has little effect on leukocyte seeding of the skin, inguinal adipose, and perigonadal adipose tissue, at rest. Furthermore, alterations in the levels of neutrophils, mast cells, macrophages, eosinophils, and blood vessels seem unlikely to explain the difference in subcutaneous adipocyte size between WT and CXCR2 KO female mice.

### 3.5 Adipocytes express CXCR2, show reduced mRNA levels of adipogenesis associated genes in CXCR2 KO mice and reduced differentiation in the presence of a CXCR2 inhibitor

**Figure 5:**
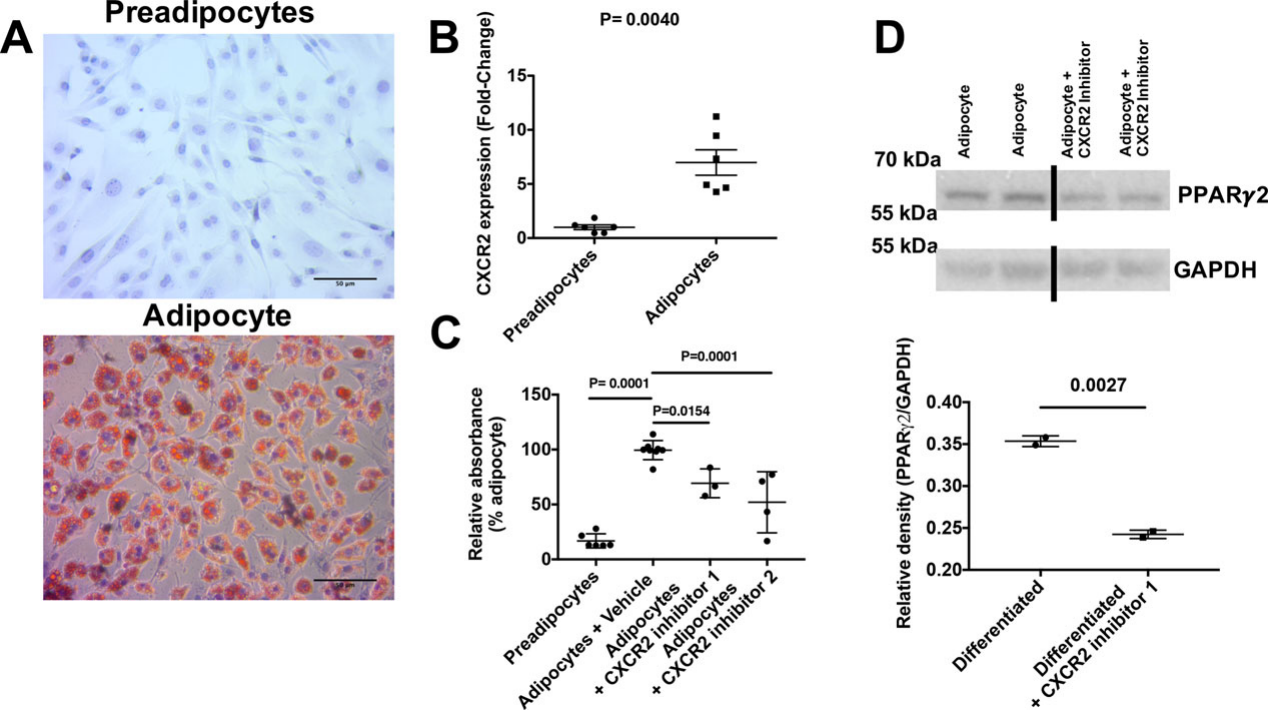
**Differentiated adipocytes and fat depots express CXCR2**. (A) 3T3‐L1 cells were differentiated into adipocytes and cells stained with oil red‐O (scale bars: 50 μm). (B) mRNA was extracted before and after differentiation, reverse transcribed to cDNA and analyzed for CXCR2 expression relative to the house‐keeping gene 18s. (C) Oil red‐O staining was quantified in undifferentiated and differentiated adipocytes in the absence or presence of two different CXCR2 inhibitors (expressed relative to differentiated cells). (D) Adipocytes differentiated in the absence and presence of a CXCR2 inhibitor 1 were analyzed for PPARγ protein expression relative to GAPDH levels (left panel) and quantified relative to GAPDH using densitometry. Data are plotted as mean (±sem), where each symbol represents an experimental replicate. Analyzed using an unpaired *t* test with Welch's correction (B and D) or one‐way ANOVA with TUKEY's multiple comparison test (C). Data are representative of two separate experiments

Previous in vitro studies have reported that adipocytes themselves express CXCR2 and that it plays a role, in combination with its ligands, in their development and size.^24^, ^32^, ^33^, ^34^ To confirm this expression pattern, we differentiated adipocytes from pre‐adipocytes, which was confirmed by positive oil red‐O staining of the differentiated cells (Fig. 5A). qRT‐PCR analysis of these cells pre, and post, adipocyte differentiation revealed increased expression of CXCR2 in the differentiated cells (Fig. 5B). Flow cytometry revealed that preadipocytes and differentiated adipocytes express CXCR2 protein (Supplemental Fig. 1A). Quantification of oil red‐O staining demonstrated a significant reduction in the presence of two different CXCR2 inhibitors during adipocyte differentiation (Fig. 5C), compared to differentiated cells alone, associated with a reduction of PPARγ expression (Fig. 5D and Supplemental Fig. 1B). Inhibitor 2 seemed to show a greater ability to inhibit adipogenesis; however, this may be associated with its effect on viability of these cells not seen with inhibitor 1 (Supplemental Fig. 1C).

### 3.6 Adipogenesis‐related genes are downregulated in adipose tissue of CXCR2 KO mice

**Figure 6:**
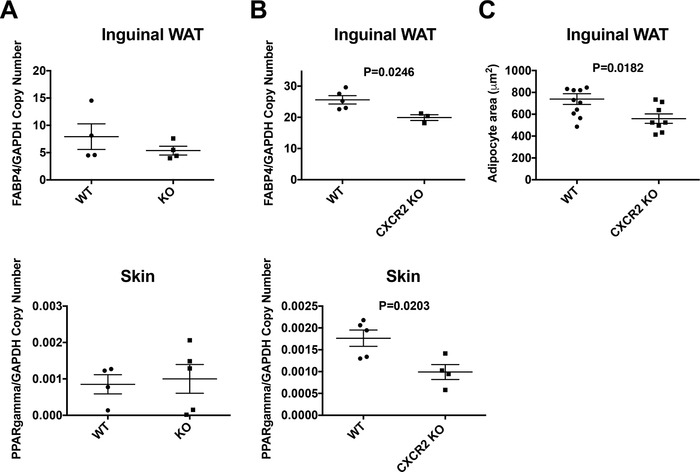
**Adipogenesis related genes are down‐regulated in the skin and inguinal adipose tissue of juvenile CXCR2 KO mice**. mRNA was extracted from inguinal adipose tissue and the skin of (A) adult and (B) juvenile female wild‐type and CXCR2 KO mice and analyzed for expression of PPARγ and FABP4 relative to the house‐keeping gene GAPDH. (C) Inguinal adipose tissue from juvenile mice was dissected, fixed, processed, and sections stained with H&E. The area occupied by individual adipocytes was calculated and plotted. Data are plotted as mean (±sem) where each symbol represents data from an individual mouse and analyzed using an unpaired *t* test. Data are representative of two separate experiments

qRT‐PCR was next used to investigate relative mRNA expression of PPARγ and fatty acid binding protein 4 (FABP4) in inguinal and subcutaneous adipose depots. Both of these genes play a key role in adipogenesis and have been shown, in vitro, to be transcriptionally regulated in response to CXCR2 signaling.^24^, ^34^ In adult mice (8‐12 wk of age) no significant differences in expression of these genes was observed in either of these adipose depots (Fig. 6A). However, when mRNA was extracted from the same adipose depots of juvenile (6 wk of age) female mice and analyzed (Fig. 6B) we found that FABP4 showed a small, but significant (*P* = 0.0246), reduction in expression in the inguinal fat depot of these juvenile mice and that there was a 50% reduction in the levels of PPARγ mRNA in the skin of juvenile mice (Fig. 6B). Analysis of inguinal adipocytes from juvenile mice demonstrated that there was a significant difference in adipocyte size between WT and CXCR2 KO females (Fig. 6C). However, inguinal adipocytes from WT juvenile mice (6 wk old) were smaller than those in adults, whereas there was little difference in adipocyte size between 6 and 8 wk in the CXCR2 KO mice (Figs. 2 and 6C). This suggests that at around 6 wk of age adipocytes in the inguinal fat depot increase in size during transition to adulthood, a time period key to inguinal mammary gland maturation,^12^ and it is at this stage that CXCR2 may play a role in inguinal adipogenesis. This would possibly confirm findings from a previous study demonstrating a role for CXCR2 in adipogenesis at specific points of maturation.^24^, ^34^

Taken together these data demonstrate that CXCR2 KO mice show a significant reduction in expression of genes that are key to adipogenesis at 6 wk of age in specific fat depots. The specificity and timing of adipogenesis‐related gene expression suggests that CXCR2 is involved in the development of different adipose depots at specific phases of mouse development.

These data, in combination with previous in vitro mechanistic studies,^24^, ^29^, ^32^, ^34^ demonstrate that CXCR2 plays a key role in adipogenesis and regulates adipocyte cell and depot size in vivo, possibly via its direct expression on adipocytes.

## 4 DISCUSSION

In this study, we have demonstrated a role for the chemokine receptor CXCR2 in adipocyte cell development and thus adipose depots, in vivo. Our data, in combination with previous detailed mechanistic in vitro^24^, ^32^, ^34^ and in vivo^29^ studies, suggest that the role of CXCR2 in resting adipose development is due to its expression on adipocytes themselves, and is therefore independent of neutrophils and their well‐characterized pro‐inflammatory functions.^1^ Whereas the full range of adipogenesis related genes controlled by CXCR2 signaling, and the phase of maturation at which they function, remain to be determined, it is clear that chemokine signaling contributes to the regulation of adipogenesis. This may be in a cell/context specific manner^24^ mediated through ERK and JNK phosphorylation^24^, ^34^ and by the regulation of expression of adipogenesis‐related genes such as PPARγ and FABP4.

This CXCR2 adipogenesis function represents a novel, and noninflammatory, role for this chemokine receptor and widens our understanding of its role in biology. Several other chemokine receptors have been associated, both directly and indirectly, with regulation of adipose tissue. CCR2 and CX3CR1 have been implicated in adipose regulation via their effects on macrophage infiltration into adipose tissue and the subsequent regulatory role of these cells.^30^, ^31^ CXCR4 is expressed on adipocytes and limits obesity, demonstrated by CXCR4 adipocyte‐specific KO mice displaying exaggerated high fat diet‐induced obesity, compared to WT mice.^35^ This study suggested that CXCR4 played a role in limiting inflammatory cell infiltration into adipose tissue but also in the thermogenic activity of brown adipose tissue. Additionally, CXCR7 activation has been shown to limit atherosclerosis by regulation of blood cholesterol.^36^ Thus, CXCR2 represents one facet of chemokine mediated control of adipose tissue and related diseases; however, it seems to function at rest via direct adipogenesis effects and not through regulation of inflammatory cells entering into adipose tissues.

This significant effect on a fundamental process such as adipocyte development may provide mechanistic clues to previous phenotypes associated with CXCR2 KO mice. CXCR2 and its ligand CXCL5 are enriched in human atherosclerotic coronary arteries, where a CXCL5 genetic variant may be a molecular marker and target for treatment in coronary artery disease.^37^ Furthermore, CXCR2 KO mice have improved sensitivity to insulin in an obesity induced model of insulin resistance.^25^ Given the significant role that adipocytes play in atherosclerosis and obesity induced insulin resistance^38^ it seems possible, though speculative at this stage, that CXCR2 mediated adipocyte differentiation may play a significant role in these processes.

In summary we have shown that CXCR2 KO mice have smaller subcutaneous fat depots due to smaller individual adipocytes, a finding similar to adipocytes from other adipose depots. This seems to be due to reduced expression of key adipogenesis‐related genes at specific time points in the life cycle of the mouse. Our study, therefore, reveals an atypical function for CXCR2 and contributes to our overall understanding of the regulation of adipocity by chemokines and their receptors.

## DISCLOSURES

The authors declare no conflicts of interest.

## Supporting information

Supplementary Fig. 1
