# Supplementary figures and images for: The chemokine receptor CXCR2 contributes to murine adipocyte development
Source: J Leukoc Biol. 2018 Dec 5;105(3):497–506. doi: 10.1002/JLB.1A0618-216RR (PMC6392114; doi:10.1002/JLB.1A0618-216RR)

# Supplementary Fig. 1

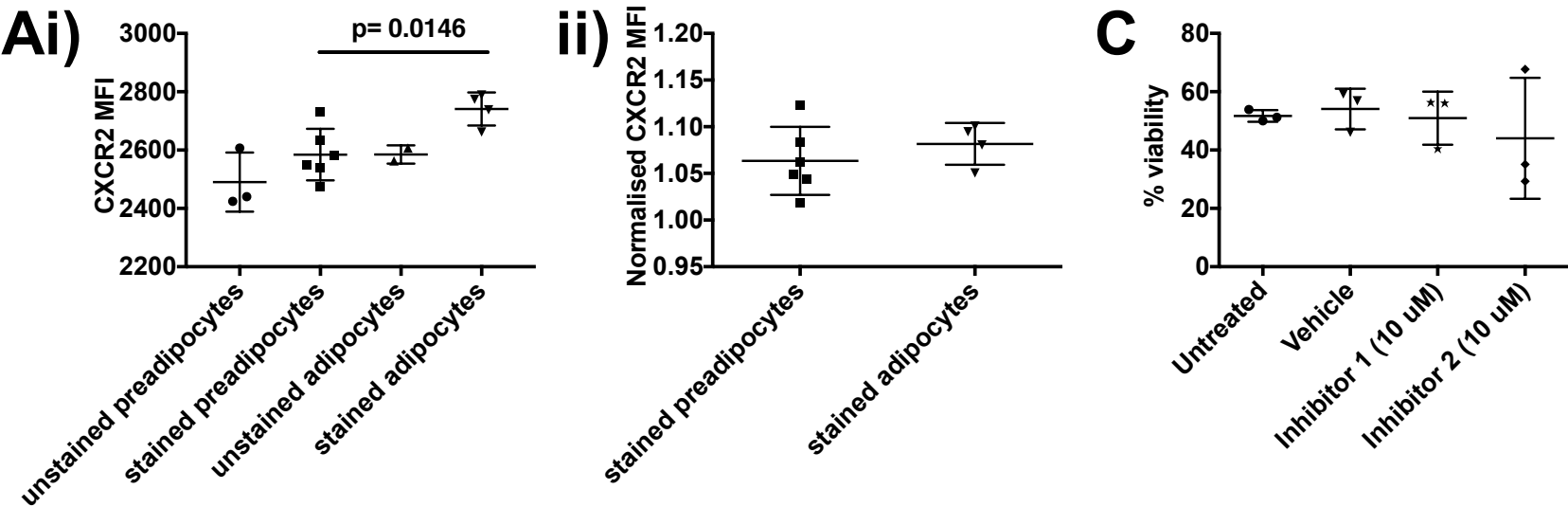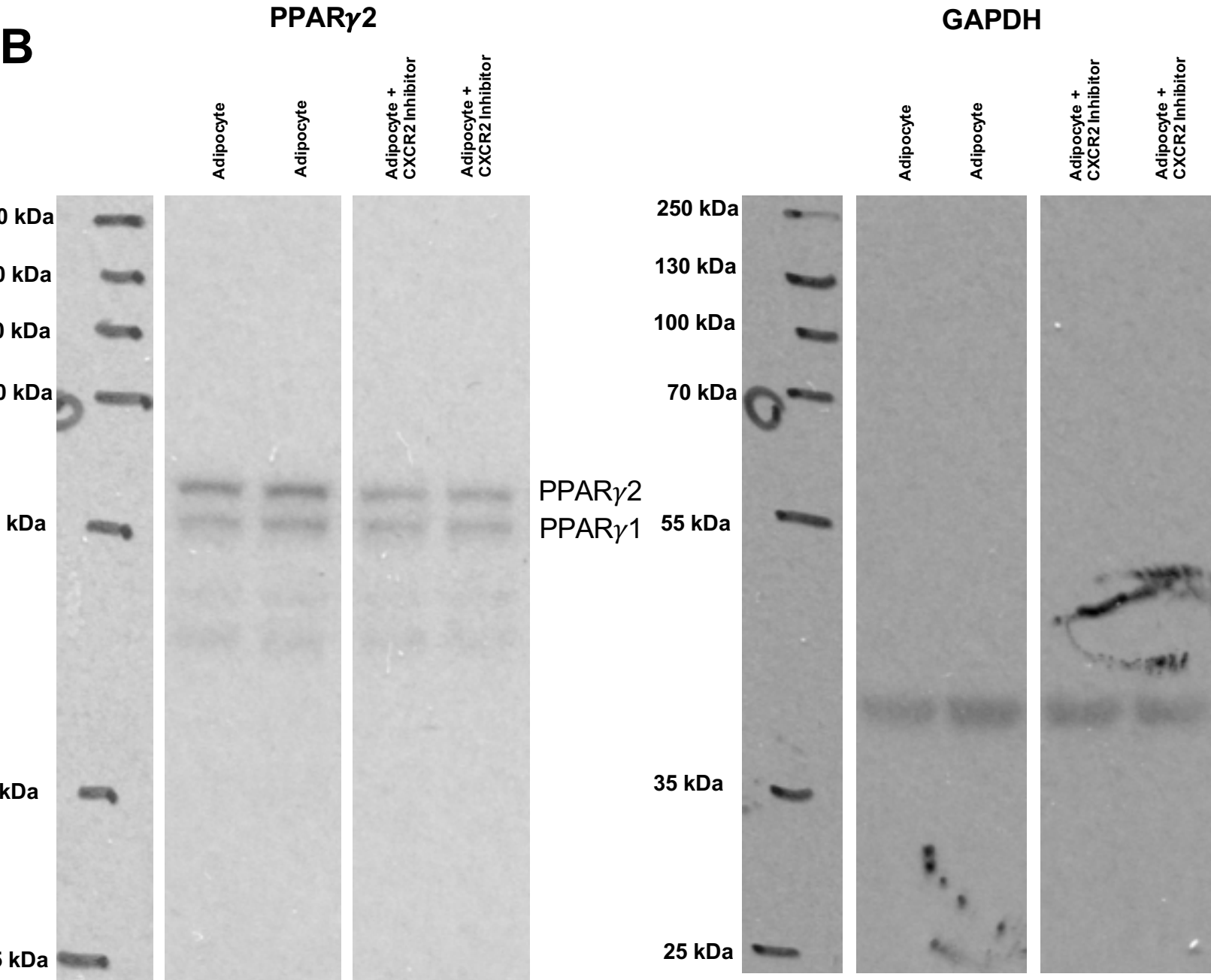

Supplement: Supplementary file 1 — Supplementary Fig. 1 [file JLB-105-497-s001.pdf]
